# Stiffness and Frequency Response Characteristics of Glass Fiber Reinforced Plastic Wave Springs with Different Periods and Its Finite Element Analysis

**DOI:** 10.3390/ma15249045

**Published:** 2022-12-17

**Authors:** Xianglong Wen, Kai Fu, Yukuan Dou, Xu Xia, Jinguang Zhang

**Affiliations:** 1 School of Mechanical and Electronic Engineering, Wuhan University of Technology, Wuhan 430070, China; 2 Hubei Provincial Engineering Technology Research Center for Magnetic Suspension, Wuhan 430070, China; 3 Sanya Science and Education Innovation Park of Wuhan University of Technology, Sanya 572000, China; 4 Institute of Advanced Material and Manufacturing Technology, Wuhan University of Technology, Wuhan 430070, China

**Keywords:** GFRP, stiffness, wave spring, finite element analysis

## Abstract

Based on the stiffness theory of wave spring, this paper proposes the wave springs made of glass fiber reinforced plastic (GFRP) and investigates the effect of the number of periods on the GFRP wave springs’ stiffness and frequency response characteristics. First of all, five different periods of composite wave springs which have identical outside dimensions are designed. Afterwards, the load-displacement curves of the GFRP wave springs are obtained using a combination of experimental and finite element analysis (FEA). Finally, the frequency response characteristics of the GFRP wave springs are measured using a force hammer excitation, and the experiment results of a GFRP wave spring are compared with a metal helical spring. The results show that the stiffness of the GFRP wave spring decreases from 34.84 N/mm to 20.59 N/mm with the increase in the number of periods. As the number of periods increases, the vibration attenuation increases from 16.32 dB to 69.17 dB. The stiffness of the GFRP wave spring is increased by 90.30% and the weight is reduced by 26.78%. The vibration isolation interval and vibration attenuation amplitude of the GFRP wave spring are higher than the metal helical spring.

## 1. Introduction

Spring is an elastic body whose function is to store energy when compressed and release the stored energy when it returns [1]. To save energy and improve the performance of vibration isolation systems, advanced springs with light weight and high performance have been used for automotive and mechanical systems. Because of their high specific strength, more elastic strain storage capacity, excellent corrosion resistance and damping properties, fiber reinforced composites have gradually replaced steel or conventional metals and have become a key material for making advanced springs in recent years [2].

Currently, most of the research focuses on the materials, mechanical properties and vibration characteristics of springs. Material properties will directly affect the performance, process feasibility and manufacturing cost of composite springs. Therefore, choosing the right material is the primary issue in composite spring design. Some scholars have used glass fiber-reinforced polymer to reinforce tubular T/Y joints, and the results show that glass fiber reinforced plastic (GFRP) significantly improves the flexibility of tubular joints [3]. The suitability of steel, S-glass fiber and carbon fiber prepreg composites as automotive suspension springs has been investigated in related literature. It was found that although the load bearing capacity of fiber-reinforced composite helical springs is lower than the steel springs, but the specific strength of composite springs is higher [4]. Part of the literature investigates the feasibility of replacing metal helical springs with composite helical springs. It was found that by using a composite helical spring with a rubber core and a woven outer layer, the load capacity and stiffness of the helical spring were increased [5,6]. The related literature gives the results of stiffness and damping predictions using finite element methods on the basis of laminated, corrugated, metal-polymer composites. It is concluded that decreasing the thickness of the viscous polymer layer will increase the damping and stiffness [7]. Springs are often subjected to impact loading, and the damage of fiber-reinforced polymer matrix composite materials induced by impact load is one of the most critical factors that restrict extensive use of these materials. Therefore, the material, geometry, event- and environment-related conditions that affect the structural behavior of fiber-reinforced polymer matrix composites to impact loading is discussed [8]. It was found that the use of helical springs made of composite materials is problematic in some cases because significant torsional stresses are generated and these stresses cause delamination of the helical springs [9]. From the above studies, it can be seen that process defects can seriously affect the performance of the structure. Relevant literatures have studied the effects of laser beam power, welding speed, blank holder force (BHF), material properties and friction coefficient on laser welding blanks. In addition, the laser welding and deep drawing processes were numerically simulated using Simufact Welding and Abaqus/Explicit software, with optimal points of 1.15 mm and 0.21 KJ for weld line displacement and absorbed energy, respectively. Additionally, an automated real-time computer-aided inspection (CAI) method and distortion analyses of aluminum laser welded blanks (LWBs) using a 3D laser scanner are presented, along with experimental validation that the proposed method identified defects on flange area in the range of ±3 mm, which enables rejecting parts out of tolerances before succeeding to the next step in the value chain [10,11].

Several scholars have conducted studies on the vibration characteristics of composite helical springs. Timoshenko beam theory was used to derive the control equations and to study the dynamic characteristics of arbitrarily shaped composite helical springs [12]. The study not only theoretically models the free vibration problem of unidirectional composite cylindrical helical springs, but also investigates the effect of the number of coils, the helix pitch angle, the ratio of the minimum to maximum cylindrical radius and the ratio of the maximum cylindrical diameter to the wire diameter on the free vibration frequency of composite helical springs [13,14]. Some scholars have studied the free vibration behavior of cylindrical helical springs of laminated composite materials with rectangular cross-section and derived the differential equations of motion for the springs consisting of 14 first-order partial differential equations using natural bending and twisted anisotropic beam theory [15,16,17,18]. Some scholars proposed to use the stiffness matrix method based on the first-order shear deformation theory to predict the basic natural frequency and buckling load of the non-cylindrical unidirectional composite spiral spring under the initial static axial force and bending moment [19,20]. A method for calculating the intrinsic frequency of a carbon epoxy helical spring is presented in the related literature. The method describes the linear dynamical behavior of a composite excitation helical spring [21]. The modal model of a five-degree spring-mass vibration system has also been predicted by using the state-space method [22].

At present, the main research object of fiber-reinforced composite spring is helical spring, and there is little research on composite wave springs. Composite helical springs are usually prepared using pultrusion and fiber-winding technology processes [23], while GFRP wave springs can be prepared by using autoclaves. The preparation process of GFRP wave spring is simple, and the mold can be reused, which is suitable for mass production. In addition, the helical spring mainly bears the torsional load, and the shear modulus is the main factor affecting its stiffness, while the wave spring mainly bears the bending load, and the modulus in the fiber direction is the main factor affecting its stiffness. For fiber-reinforced composites, the modulus in the fiber direction is generally 1–2 orders of magnitude higher than the shear modulus [24]. Composite wave springs are more in line with the current design requirements of high stiffness and low mass of composite springs [2]. In addition, the spring is a kind of damping unit, and research on the frequency response characteristics of the spring is also necessary.

This paper focuses on the effect of the number of periods on the stiffness and vibration characteristics of composite wave spring. Firstly, based on the stiffness theory of wave springs, five kinds of GFRP wave springs with different periods are designed. Then, GFRP wave springs are prepared by the autoclave process, and a combination of experiments and finite element simulations is used to obtain the load-displacement curves of the composite wave springs. Finally, the frequency response characteristics of the composite wave springs are studied by the force hammer excitation method, and the vibration isolation intervals and attenuation amplitude of the GFRP wave springs are obtained. The paper also compares the experiment results of the GFRP wave spring with the metal helical spring.

## 2. Structure Design and Fabrication of GFRP Wave Springs

### 2.1. Material

E-glass fiber is a kind of safe composite material when subjected to low stress and low strain working conditions [25]. In this paper, E-glass fiber prepreg (UD715, supplied by Jiangsu Jiu Ding New Material, China.) is selected to prepare the wave spring, whose matrix is YPH-629 epoxy resin and reinforcement is EC600 continuous glass fiber yarn. The properties of E-glass fiber prepreg are shown in Table 1.

### 2.2. Structural Design of GFRP Wave Springs

**Figure 1:**
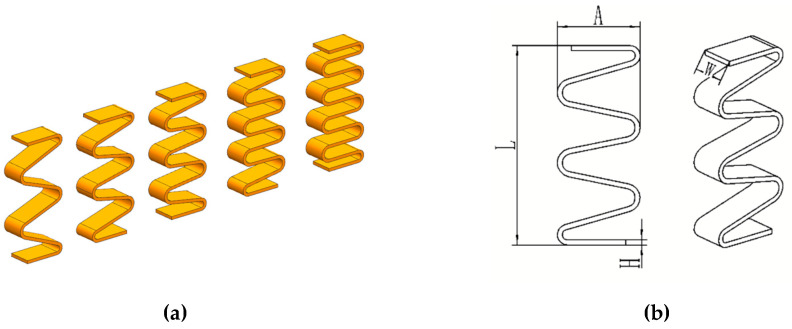
Structural diagram of GFRP wave springs. (**a**) The five different specimens, (**b**) Schematic diagram of the structure of the specimen.

This paper focuses on the effect of the number of periods on the stiffness and frequency response characteristics of GFRP wave springs. Therefore, GFRP wave springs with periods T = 2, T = 2.5, T = 3, T = 3.5 and T = 4 are designed. Figure 1 shows the structure diagram of composite wave springs, where L = 154 mm is the wave spring free height, A = 64 mm is the wave spring amplitude, H = 4 mm is the wave spring thickness and W = 34 mm is the wave spring width.

### 2.3. Fabrication of GFRP Wave Springs

**Figure 2:**
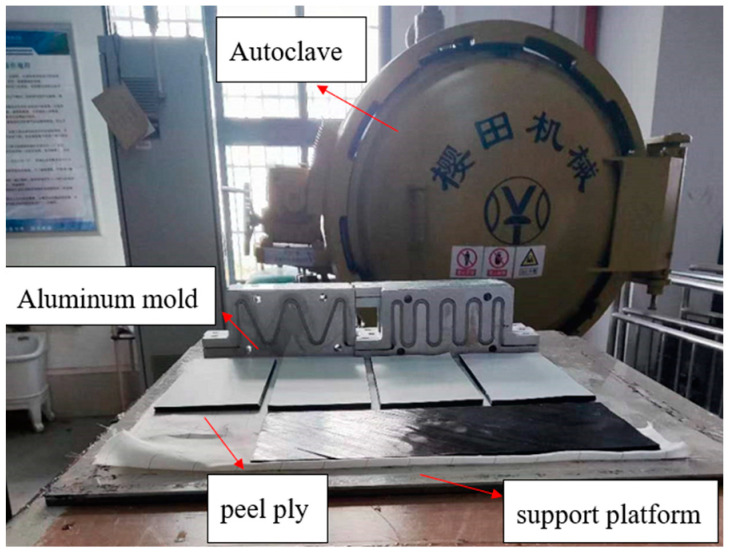
Preparation process of GFRP wave springs.

The GFRP wave springs are fabricated using the autoclave molding process [27,28]. Unlike the fabrication process of composite helical springs, the fabrication process of GFRP wave springs are simple and the molds are reusable. The fabrication process of the GFRP wave springs are as follows (Figure 2): The prepreg fabric is cut to the required size using a computer numerical control cutting machine, and the prepreg is laid onto the mold layer by layer.

The mold surface is covered with release cloth and release grease for subsequent release of specimens. Next, the mold is placed on the autoclave support platform and a breathable felt and vacuum bag are laid on the mold, and the vacuum bag is connected to the support platform using sealant for sealing. Next, push the support platform into the autoclave for high-temperature and high-pressure curing molding.

**Figure 3:**
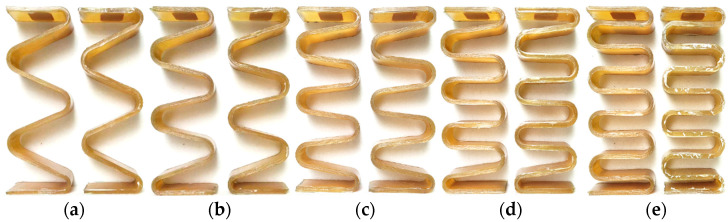
Schematic diagram of the GFRP wave spring specimens, (**a**) T = 2, (**b**) T = 2.5, (**c**) T = 3, (**d**) T = 3.5, (**e**) T = 4.

The design thickness of the specimen is 4 mm, the thickness of the single layer of glass fiber prepreg is 0.15 mm and the number of layers for each specimen is 30 layers with full zero degree for laying. Two valid specimens are made for each program (Figure 3). The dimensions of the specimens are as follows: free height L = 152.30 mm, amplitude A = 64.14 mm, thickness H = 4.20 mm and width W = 33.10 mm. There is the error between the design and actual size, which is caused by the machining tolerances of the mold and fabrication error. During the high-pressure molding step in the preparation process, the resin will flow along the width direction during the preparation of the specimen, resulting in excessive local resin content at the two end regions of the specimen. After the specimen preparation was completed, the specimen was secondary processed, and the resin-enriched area at the end of the specimen was polished.

## 3. Theoretical Calculation of GFRP Wave Spring

**Figure 4:**
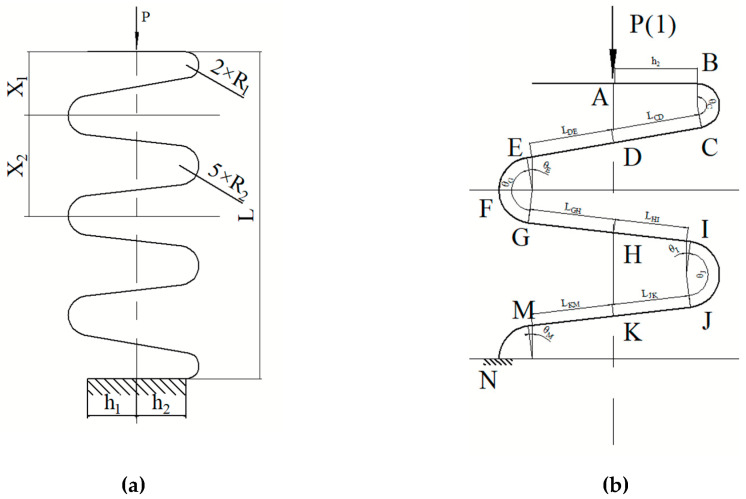
Force analysis of GFRP wave spring. (**a**) Schematic diagrams of composite wave spring, (**b**) Force analysis of single-period composite wave spring.

The development of the load-displacement response of the GFRP wave spring begins with the analysis of wave spring from isotropic materials. The force analysis of GFRP wave spring is shown in Figure 4.

Considering that the composite wave spring is a symmetrical structure and its internal force is symmetrical under the applied load, the wave spring can be divided into two parts: the end deformation area X_1_ and the middle periodic structure deformation area X_2_. Analyzing X_1_ and X_2_, the vertical displacement at point A can be calculated. Multiplying the result by the corresponding period, the displacement of the entire spring can be obtained.

According to the unit load method [29,30], in order to obtain the displacement in the vertical direction at point A, a unit force in the same direction as load P must be applied at point A, as shown in the Figure 4, in which the internal force equation of the wave spring is as follows

The bending moment from *A* to *B* is as follows:

(1) $$M_{AB}(x)=-Px,x\in (0,h_{2})$$

where, $M_{AB}$ is the bending moment of *AB* segment under the applied load, *P* is the applied load, and *h*_2_ is the length of *AB* segment.

The bending moment from *B* to *C* is as follows:

(2) $$M_{BC}(\theta )=-P(h_{2}+R_{1}\sin(\theta )),\theta \in (0,\theta_{C})$$

where $M_{BC}$ is the bending moment of *BC* segment, and *θ_C_* is the radian of *BC* segment.

The bending moment from *C* to *D* is as follows:

(3) $$M_{CD}(x)=-P(h_{2}+R_{1}\sin(\theta_{C}))(1-\frac{1}{L_{CD}}x),x\in (0,L_{CD})$$

where $M_{CD}$ is the bending moment of *CD* segment, and *L_CD_* is the length of the *CD* segment.

The bending moment from *D* to *E* is as follows:

(4) $$M_{DE}(x)=P(h_{1}+R_{2}\sin(\theta_{E}))\frac{x}{L_{DE}},x\in (0,L_{DE})$$

where $M_{DE}$ is the bending moment of *DE* segment, and *L_DE_* is the length of the *DE* segment.

The bending moment from *E* to *F* is as follows:

(5) $$M_{EF}(\theta )=P(h_{1}+R_{2}\sin(\theta )),\theta \in (\theta_{E},\frac{\pi }{2})$$

where $M_{EF}$ is the bending moment of *EF* segment.

The bending moment from *F* to *G* is as follows:

(6) $$M_{FG}(\theta )=P(h_{1}+R_{2}\sin(\theta )),\theta \in (\frac{\pi }{2},\theta_{G})$$

where $M_{FG}$ is the bending moment of *FG* segment, and *θ_G_* is the radian of *EG* segment.

The bending moment from *G* to *H* is as follows:

(7) $$M_{GH}(x)=P(h_{1}+R_{2}\sin(\theta_{G}))\frac{x}{L_{GH}},x\in (0,L_{GH})$$

where $M_{GH}$ is the bending moment of *GH* segment, and *L_GH_* is the length of the *GH* segment.

The bending moment from *H* to *I* is as fllows:

(8) $$M_{HI}(x)=-P(h_{2}+R_{1}\sin(\theta_{I}))(1-\frac{1}{L_{HI}}x),x\in (0,L_{HI})$$

where $M_{HI}$ is the bending moment of *HI* segment, and *L_HI_* is the length of the *HI* segment.

The bending moment from *I* to *J* is as follows:

(9) $$M_{IJ}(\theta )=-P(h_{2}+R_{1}\sin(\theta )),\theta \in (\theta_{I},\theta_{J})$$

where $M_{IJ}$ is the bending moment of *IJ* segment, and *θ_J_* is the radian of *IJ* segment.

The bending moment from *J* to *K* is as follows:

(10) $$M_{JK}(x)=-P(h_{2}+R_{1}\sin(\theta_{J}))(1-\frac{1}{L_{JK}}x),x\in (0,L_{JK})$$

where $M_{JK}$ is the bending moment of *JK* segment, and *L_JK_* is the length of the *JK* segment.

The bending moment from *K* to *M* is as follows:

(11) $$M_{KM}(x)=P(h_{1}+R_{2}\sin(\theta_{M}))\frac{x}{L_{KM}},x\in (0,L_{KM})$$

where $M_{KM}$ is the bending moment of *KM* segment, and *L_KM_* is the length of the *KM* segment.

The bending moment from *M* to *N* is as follows:

(12) $$M_{MN}(\theta )=P(h_{1}+R_{2}\sin(\theta )),\theta \in (\theta_{M},\frac{\pi }{2})$$

where $M_{MN}$ is the bending moment of *MN* segment.

The bending moment ${\bar{M}}_{AF}$ and ${\bar{M}}_{FN}$ of the wave spring under unit load could be obtained while *P* = 1.

According to Mohr’s theorem [31,32], the displacement of end *A* in the vertical direction is as follows:

(13) $$\Delta_{A}=\frac{2}{EI}\int_{AF}M_{AF}{\bar{M}}_{AF}dx+\frac{\left(T-1\right)}{EI}\int_{FN}M_{FN}{\bar{M}}_{FN}dx$$

where *E* is Young’s modulus, *I* is moment of inertia. When a composite material is used, the isotropic assumptions are no longer valid. In a composite material, the laminate bending moduli and Poisson ratio are as follows:

(14) $$E_{x}=\frac{12(D_{11}D_{22}-D_{12}^{2})}{t^{3}D_{22}}$$

(15) $$E_{y}=\frac{12(D_{11}D_{22}-D_{12}^{2})}{t^{3}D_{11}}$$

(16) $$G_{xy}=\frac{12(D_{66})}{t^{3}}$$

(17) $$\nu_{xy}=\frac{D_{11}}{D_{22}}$$

where *D*_11_, *D*_12_, D_22_ and *D*_66_ are coefficients from the bending stiffness matrix found from the classical lamination plate theory. In the orthotropic laminates, the elastic modulus, *E*, depends on the angle, *θ* [33] by the following relationship:

(18) $$E(\theta )={\left[\frac{m{\left(\theta \right)}^{2}}{E_{x}^{}}\left(m{\left(\theta \right)}^{2}-n{\left(\theta \right)}^{2}v_{xy}^{}\right)+\frac{n{\left(\theta \right)}^{2}}{E_{y}^{}}\left(n{\left(\theta \right)}^{2}-m{\left(\theta \right)}^{2}v_{yx}^{}\right)+\frac{m{\left(\theta \right)}^{2}n{\left(\theta \right)}^{2}}{G_{xy}^{}}\right]}^{-1}$$

where *m(θ)* is cos *θ* and *n*(*θ*) is sin *θ*.

The formula for calculating the stiffness of the GFRP wave spring is as follows [34]:

(19) $$K=\frac{P}{\Delta_{A}}$$

## 4. Experimental and FEA

### 4.1. FEA of GFRP Wave Springs

**Figure 5:**
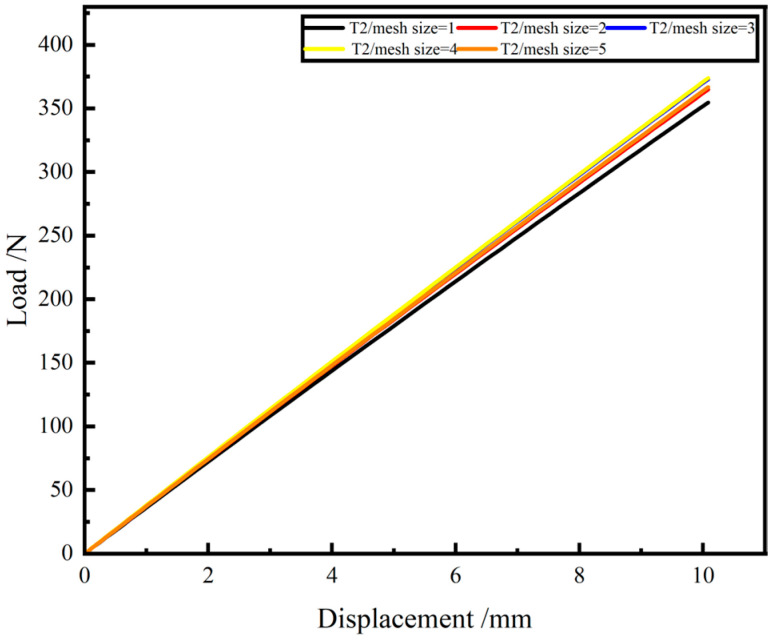
Stiffness simulation results for different mesh sizes.

In the finite element (FE) analysis, the simulation results are highly dependent on the mesh size. In this paper, the mesh sensitivity analysis of the FE model with a period of 2 are carried out. With other settings unchanged, the FE models with mesh sizes of 1 mm, 2 mm, 3 mm, 4 mm and 5 mm are analyzed and the results are shown Table 2 and Figure 5.

The difference between the simulation results with different mesh sizes does not exceed 5.5%, so the effect of the mesh size on the simulation stiffness is not significant. Generally, the smaller the mesh size, the closer the simulation results are to the actual situation, but considering the simulation time, the mesh size cannot be reduced indefinitely. Therefore, the mesh size of 1 mm is used to simulate the GFRP wave springs.

**Figure 6:**
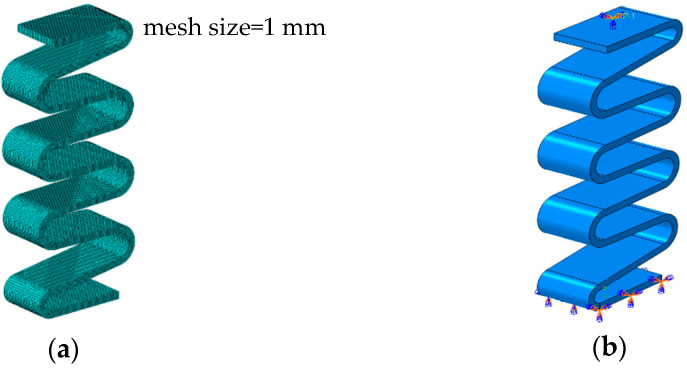
Schematic diagram of finite element simulation analysis model setting, (**a**) Finite element model of wave spring, (**b**) Simulation boundary condition settings.

Based on the structure shown in Figure 1, the corresponding 3D model of the GFRP wave springs are created by the 3D modeling software and imported into the finite element simulation software. The mesh type of the GFRP wave spring is a hexahedral mesh SC8R with an overall mesh size of 1 mm, which is divided into a total of 72,840 meshes. The material parameters used in the simulations are shown in Table 1. During the static analysis, the calculations are performed using the static general analysis step (Static, General). The boundary constraint is to fix one end of the wave spring and to couple the other end face to the reference point. Then, a linear displacement with time is applied at the reference point. Finally, the displacement and reaction force in the vertical direction at the reference point (RP-1) are output to obtain the load-displacement curve. The setup is shown in Figure 6.

### 4.2. The Experiment of Stiffness

**Figure 7:**
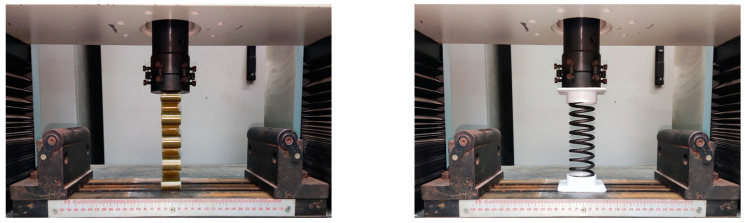
Compression performance experiment bench.

This paper uses electronic universal testing machine to experiment the stiffness performance of GFRP wave springs and metal helical spring, and the installation is shown in Figure 7.

The experiment method is compression experiment, in which the indenter moves down at a rate of 5 mm/min and the sampling frequency is 100 Hz. The experiment ends when the loaded displacement reaches 10 mm, the load-displacement data of the experiment is exported, and the weight of the composite wave springs and metal coil springs is measured.

Comparing the results of GFRP wave spring with a period of 4 (with 8 peaks and valleys) with a metal helical spring with a period of 8. The free height, mid-diameter and wire diameter of the metal helical spring are the same as the GFRP wave spring.

### 4.3. The Experiment of Frequency Response Characteristics

The experiments are conducted using the hammering method [35,36] to excite the GFRP wave springs and metal spiral spring, and the acceleration response signal is measured by an accelerometer. Fast Fourier transform (FFT) of the signal is performed to obtain the frequency domain signal and analyze the vibration characteristics of the GFRP wave springs. The specific parameters of the equipment used for the experiment are shown in Table 3.

**Figure 8:**
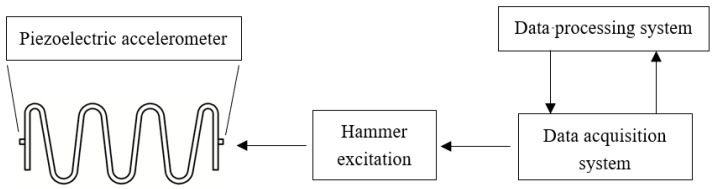
Frequency response characteristic experiment process block diagram.

The block diagram of the frequency response characteristics experiment process is shown in Figure 8. In the free state, the force hammer 8206-002 is used to generate the excitation signal for the specimen. Two B&K 4507Bx acceleration sensors acquire the input and output acceleration response signals of GFRP wave spring and input them into the B&K data acquisition system. The computer software B&K connect completes data acquisition and analysis.

**Figure 9:**
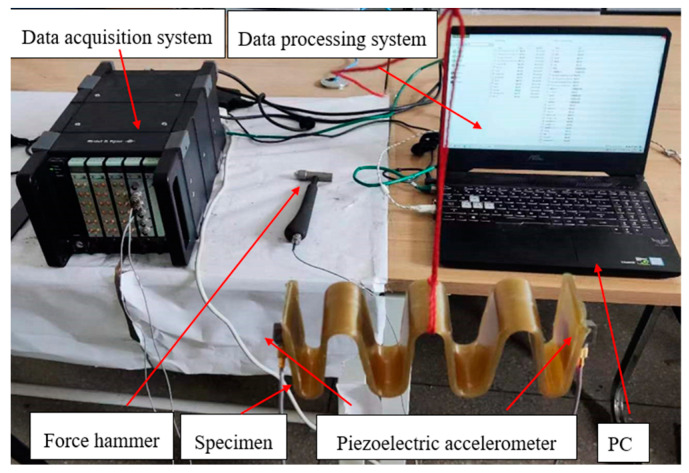
Hammer experiment bench installation diagram.

The experiment stand is set up as shown in Figure 9.

The specimen is lifted on the metal stand, and the force hammer is used to excite one end of the specimen, and two acceleration sensors are installed at each end of the specimen to collect the input and output acceleration signals. Then the signals collected by the accelerometers are transferred to the data processing system through the data acquisition system. The data processing system is used to read and save the data.

## 5. Results and Discussion

### 5.1. The Result of Stiffness

#### 5.1.1. FEA and Experimental Results on the Stiffness of the GFRP Springs

**Figure 10:**
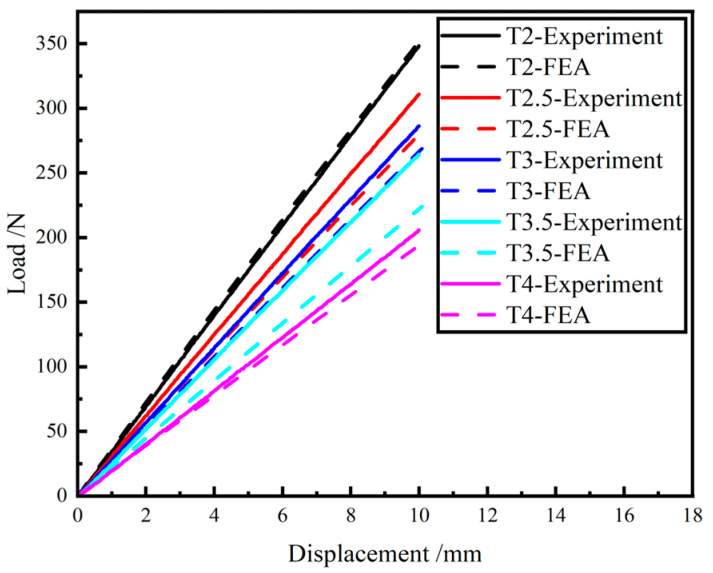
Load-displacement curve of simulation and experiment.

The load-displacement curves obtained by simulation are compared with the curves that obtained from experiment, as shown in Figure 10 and Table 4.

From the results, it can be seen that the maximum error between the simulated stiffness and the experimental stiffness is 15.35%. And the trend of the simulation stiffness and the experiment stiffness is consistent, and the stiffness decreases with the increase in the period number.

From Equation (13), it can be concluded that the displacement of the GFRP wave spring increases with the increase in the number of periods for the same load. This causes the stiffness of the GFRP wave spring to decrease as the number of periods increases. From the experimental results, it can be seen that the trend of stiffness variation of the GFRP wave spring is consistent with the theoretical trend.

#### 5.1.2. Comparison of Stiffness Results of GFRP Wave Spring and Metal Helical Spring

**Figure 11:**
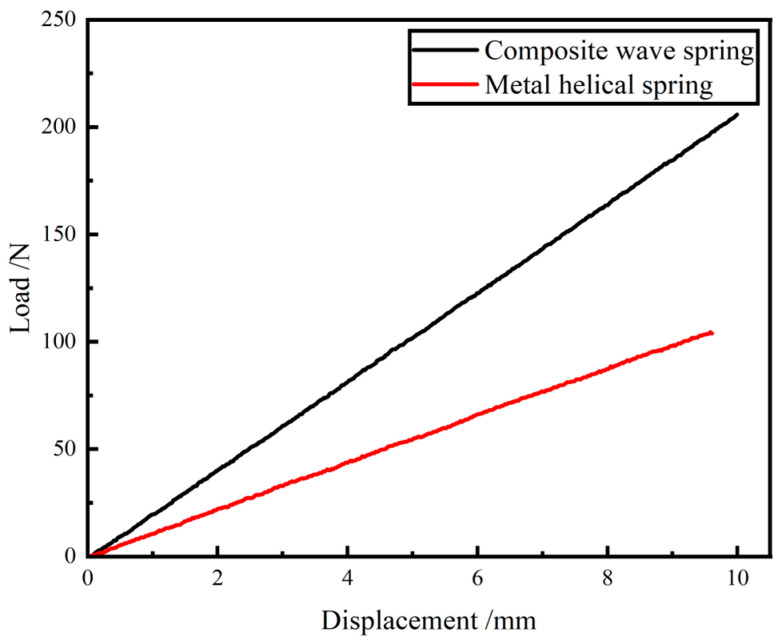
Experiment results of GFRP wave spring and metal helical spring.

The experiment results for the GFRP wave spring and the metal helical spring are shown in Figure 11.

The stiffness of the GFRP wave spring is 20.59 N/mm and the stiffness of the metal helical spring is 10.82 N/mm. The weight of the GFRP wave spring and the metal helical spring are 157.82 g and 215.65 g.

From the experiment results, it can be seen that the stiffness of the GFRP wave spring is increased by 90.30% and the weight is reduced by 26.82%.

### 5.2. Results of Frequency Response Characteristics

#### 5.2.1. Results of Frequency Response Characteristics of GFRP Wave Springs

**Figure 12:**
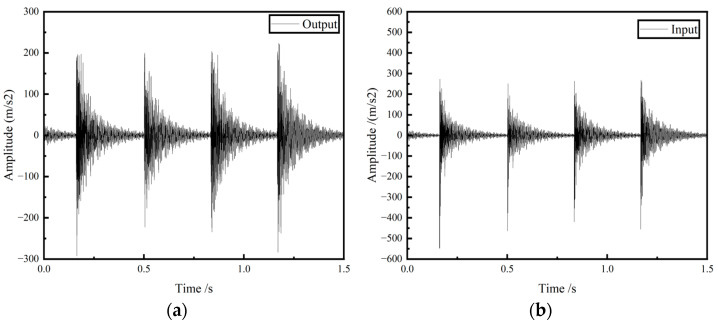
The response curves of GFRP wave springs, (**a**) time domain input signal, (**b**) time domain output signal.

After completing the frequency response experiment, the time domain signal measured by the acceleration sensor was read in the B&K connect software to obtain the vibration response curve of the GFRP wave springs. The input and output signals are shown in Figure 12.

The fast Fourier transform of the collected data is directly performed in the software B&K connect to obtain the frequency response curves of the GFRP wave springs. In order to show more intuitively the vibration isolation effect of GFRP wave springs, the FRF is used to characterize the vibration isolation effect. The FRF [37] can be obtained from Equation (20).

(20) $$FRF=20\log\frac{a_{2}}{a_{1}}$$

where *a*_2_ is the acceleration value at the output, and *a*_1_ is the acceleration value at the input.

**Figure 13:**
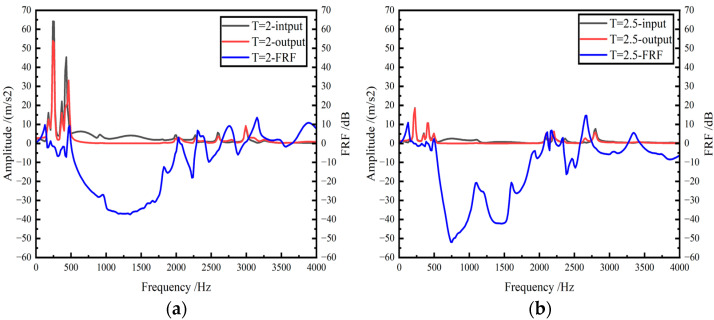

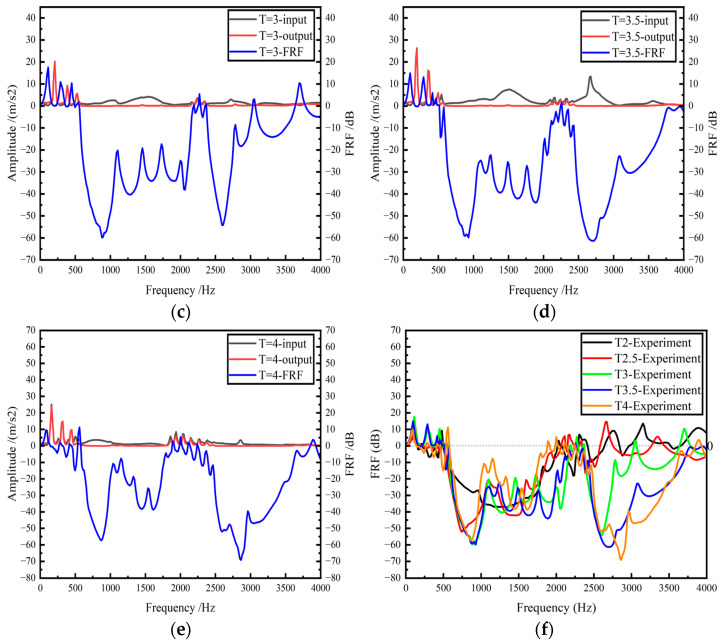
Frequency domain signals of GFRP wave springs and its FRF curve, (**a**) T = 2, (**b**) T = 2.5, (**c**) T = 3, (**d**) T = 3.5, (**e**) T = 2, (**f**) the FRF curve of GFRP wave spring with different period.

The frequency response and FRF curves of the GFRP wave springs for different periods are shown in Figure 13 and Table 5.

It can be seen from Figure 12 that the vibration isolation range of the GFRP wave spring with a period of 2 is 580–2016 Hz. When the excitation frequency is 1360 Hz, the maximum vibration attenuation amplitude reaches 37.39 dB.

For the GFRP wave spring with a period of 2.5, there are three vibration isolation intervals: 512–2064 Hz,2336–2576 Hz and 2752–3264 Hz. When the excitation frequency is 752 Hz, the maximum vibration attenuation amplitude is 51.96 dB.

For the GFRP wave spring with a period of 3, there are three vibration isolation intervals: 560–2192 Hz, 2352–3024 Hz and 3072–3632 Hz. When the excitation frequency is 880 Hz, the vibration attenuation amplitude reaches the maximum 59.85 dB.

For the GFRP wave spring with a period of 3.5, there are two vibration isolation intervals: 576–2240 Hz and 2256–3792 Hz. When the excitation frequency is 928 Hz, the maximum vibration attenuation amplitude reaches 61.26 dB.

For the GFRP wave spring with a period of 4, there are two vibration isolation intervals: 576–1920 Hz and 2256–3681 Hz. When the excitation frequency is 2864 Hz, the maximum vibration attenuation amplitude reaches 69.17 dB.

From the results, it can be concluded that wave springs with different periods have different vibration isolation effects at different excitation frequencies, and the vibration isolation effect becomes more obvious as the number of periods increases. When the excitation frequency is less than 580 Hz, the vibration isolation effect of the GFRP wave spring is poor. When the excitation frequency is 580–1920 Hz, the vibration attenuation is especially obvious, and the vibration attenuation increases gradually with the increase in the number of periods, and finally stabilizes at about 59.87 dB. When the excitation frequency is 2000–4000 Hz, with the increase in the number of periods, the vibration isolation interval of the wave springs changes from narrow to wide and less, and the vibration attenuation amplitude increases to 69.17 dB.

#### 5.2.2. Comparison of Frequency Response Characteristics Results of GFRP Wave Spring and Metal Coil Spring

**Figure 14:**
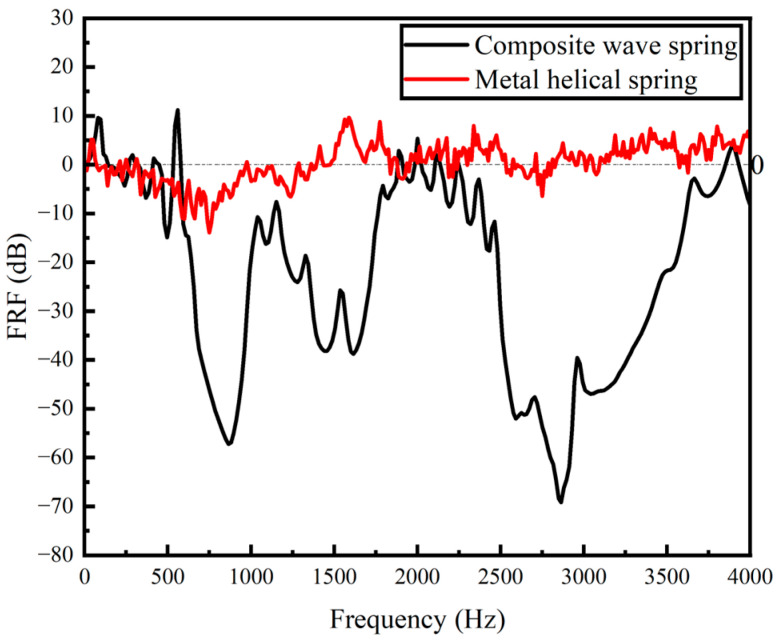
The results of frequency response characteristics of GFRP wave spring and metal helical spring.

The experiment results for the GFRP wave spring and the metal helical spring are shown in Figure 14. The vibration isolation range of the GFRP wave spring is 576–1920 Hz and 2256–3681 Hz with a maximum vibration attenuation of 69.17 dB, and the vibration isolation range of the metal helical spring is 412.5–975 Hz with a maximum vibration attenuation of 13.92 dB.

From the experiment results, it can be seen that the vibration isolation interval and vibration attenuation of the GFRP wave spring are higher than those of the metal helical spring. This is due to the fact that the composite material has better damping properties and energy storage capacity [38,39].

## 6. Conclusions

In this paper, GFRP wave springs with different periods are designed. The stiffness of the GFRP wave springs with different periods and their frequency response characteristics are studied by experiments and simulations. The main conclusions are as follows:

1. GFRP wave springs which are prepared by using the autoclave are designed.
2. The trend of the simulation and experiment results are consistent, and the stiffness of the GFRP wave spring gradually decreased as the period increased, and the maximum error between the simulation and experiment stiffness is 15.35%, and the minimum error is 1.8%.
3. As the period of the GFRP wave springs increases, the vibration isolation interval and vibration attenuation become larger, with a maximum attenuation of 69.17 dB.
4. The stiffness of the GFRP wave spring is increased by 90.30% and the weight is reduced by 26.82% compared to the metal helical spring. The vibration isolation interval and vibration attenuation of the GFRP wave spring are higher than the metal helical spring.

At present, this paper only investigates the effect of the number of periods on the GFRP wave spring stiffness and frequency response characteristics. The conclusions can be extended and applied to the design of wave springs of various fiber reinforced composite materials, and also provide a basis for subsequent research. In the future work, the factors besides period will be investigated on the stiffness and frequency response characteristics, such as size, ply angle, preparation technique and the materials. In addition, the load-bearing capacity of GFRP wave spring and its influencing factors will also be studied.

## Figures and Tables

**Table 1:** Mechanical properties of the E-glass fiber prepreg [26].

Engineering Constants	Values	Engineering Constants	Values
E_1_(GPa)	30	X_t_(MPa)	850
E_2_(GPa)	8	Y_t_(MPa)	35
E_3_(GPa)	8	Z_t_(MPa)	35
ν_12_(GPa)	0.3	X_c_(MPa)	500
ν_13_(GPa)	0.3	Y_c_(MPa)	120
ν_23_(GPa)	0.4	Z_c_(MPa)	120
G_12_(GPa)	4.5	S_12_(MPa)	104
G_13_(GPa)	4.5	S_13_(MPa)	104
G_23_(GPa)	3.8	S_23_(MPa)	70

**Table 2:** Stiffness simulation results for different mesh sizes.

Mesh Size/mm	1	2	3	4	5
Stiffness simulation N/mm	35.47	36.49	37.30	37.38	36.67
%error simulation	/	2.88%	5.16%	5.38%	3.38%

**Table 3:** Model and Parameters of Experiment Equipment.

Equipment	Model	Parameters
Piezoelectric accelerometer	4507Bx	9.688 mV/(m/s^2^)
Force hammer (Force Sensor)	8206-002	2.27 mV/N
Data acquisition system	3660-C-LAN-XI	-
Analyzing software	BK Connect	-

**Table 4:** Summary of the Stiffness results on GFRP wave springs.

Type of Springs	T = 2	T = 2.5	T = 3	T = 3.5	T = 4
Stiffness experiment-A N/mm	35.25	31.33	27.60	25.97	21.41
Stiffness experiment-B N/mm	34.43	30.85	29.66	27.11	19.77
Average value N/mm	34.84	31.09	28.63	26.45	20.59
Stiffness simulation N/mm	35.47	28.21	26.88	22.39	19.57
%error simulation	1.80%	9.26%	6.11%	15.35%	4.95%

**Table 5:** Vibration isolation interval of GFRP wave spring with different periods.

Periods	Vibration Attenuation Interval/Hz	Min of FRF/dB
T = 2	496–2048	−37.39
T = 2.5	512–2064 2336–2576 2752–3264	−51.88 −16.32 −5.7
T = 3	560–2192 2352–3024 3072–3632	−59.85 −54.15 −13.97
T = 3.5	576–2240 2256–3792	−59.88 −61.26
T = 4	576–1920 2256–3681	−57.24 −69.16

## Data Availability

The data presented in this study are available on request from the corresponding author.
